# Exposure to hepatitis C virus in homeless men in Central Brazil: a cross-sectional study

**DOI:** 10.1186/s12889-016-3952-6

**Published:** 2017-01-18

**Authors:** Priscilla Martins Ferreira, Rafael Alves Guimarães, Christiane Moreira Souza, Lara Cristina da Cunha Guimarães, Cleiciane Vieira de Lima Barros, Karlla Antonieta Amorim Caetano, Giovanni Rezza, Lila Spadoni, Sandra Maria Brunini

**Affiliations:** 1 Faculty of Nursing, Federal University of Goiás, Goiânia, Goiás Brazil; 2 University of the Federal District, Brasília, Brazil; 3 Istitute Superiore di Sanitá, Rome, Italy; 4 Faculty of Psicology, UniEvangélica, Anápolis, Goiás Brazil

**Keywords:** HCV, Epidemiology, Risk factors, Prevalence, Homeless

## Abstract

**Background:**

Homeless men are highly vulnerable to acquisition of the hepatitis C virus (HCV) compared to the general population. In Brazil, a country of continental dimensions, the extent of HCV infection in this population remains unknown. The objective of this study is to investigate the epidemiological profile of exposure to HCV in homeless men in Central Brazil.

**Methods:**

A Cross-sectional study was conducted in 481 men aged over 18 years attending therapeutic communities specialized in the recovery and reintegration of homeless people. Participants were tested for anti-HCV markers using rapid tests. Poisson regression analysis was used to verify the risk factors associated with exposure to HCV.

**Results:**

The prevalence of HCV exposure was 2.5% (95.0% CI: 1.4 to 4.3%) and was associated with age, absence of family life, injection drug use, number of sexual partners, and history of sexually transmitted infections (STI). Participants reported multiple risk behaviors, such as alcohol (78.9%), cocaine (37.1%) and/or crack use (53.1%), and inconsistent condom use (82.6%). Injection drug use was reported by 8.7% of participants.

**Conclusions:**

The prevalence of HCV infection among homeless men was relatively high. Several risk behaviors were commonly reported, which shows the high vulnerability of this population. These findings emphasize the need for the development of specific strategies to reduce the risk of HCV among homeless men.

## Background

Hepatitis C virus (HCV) infection is a major public health problem worldwide [1, 2]. It is estimated that 2.8% of the world population, which corresponds to 185 million people, is living with the chronic form of hepatitis C, and that each year about 350 thousand people die as a result of its complications, such as liver cirrhosis and hepatocellular carcinoma [2, 3]. In Brazil, the prevalence of HCV infection in the general population is 1.3%, with differences among Brazilian regions (from 0.68% in the Northeast to 2.1% in the North); in the Midwest, a prevalence of 1.6% is estimated among individuals over 20 years-old [4].

HCV is transmitted predominantly by the parenteral route, but it can also be transmitted from mother-to-child and through sexual contact [5]. Therefore, individuals who practice specific risk behaviors, such as injection and non-injection drug use, sharing needles and syringes, unprotected sex, and multiple sexual partners, are at increased risk of HCV infection [4, 6, 7]. In addition to behavioral determinants, factors related to social and programmatic vulnerabilities, such as low income and education, discrimination based on social condition or sexual orientation, loss of family ties, and difficulty accessing health services may contribute indirectly to the spread of HCV, especially in key populations (non-injection and injection drug users, sex workers, men who have sex with men, and homeless people) [8, 9].

It is estimated that each year about 60 to 70% of cases of hepatitis B and C occur in vulnerable populations, including people living on the street [8]. HCV infectionin homeless people, a population mainly composed of men [10–12], can be conceptualized by the interaction of individual, social, and programmatic vulnerabilities that expose this population to risk factors that can increase their risk for infection [9]. Injection and not injection drug use, sharing paraphernalia for drug use, tattoos, sharing of personal care items, prior incarceration, and sexual risk behaviors have been associated with HCV infection in this population [13, 14].

Studies have shown a high prevalence of HCV among homeless men, ranging from 25.1 to 34.3% in Asia [15, 16], 19.0 to 26.5% in Europe [9, 17], and 4.84 to 66.0% in North America [18, 19]. In 2012, a global systematic review and meta-analysis estimated a prevalence of 21.0% (95% CI: 13.0 to 28.0%) in homeless men [12]. In Brazil, the only study conducted in homeless men, found a prevalence of 8.5% in 330 individuals in São Paulo [11].

In Brazil, a country of continental dimensions, the extent of HCV infection among homeless men remains unknown, with the only study conducted about HCV epidemiology in this population being limited to the Southeastern region [11]. The approach to this infection requires different strategies both for diagnosis and for compliance with prevention and treatment protocols in the homeless. We believe that this study will contribute to the strengthening of public health and social policies aimed at the prevention of HCV in homeless person, since it presents important data about the epidemiological profile of the infection in this population in Brazil. In this way, the determinants of HCV exposure presented in this investigation can be taken into account in the planning and implementation of health promotion and infection prevention and comprehensive care actions, with emphasis on strengthening health education actions, availability of diagnostic tests in institutions that serve homeless persons, early treatment, provision of condoms, and epidemiological surveillance. In order to bridge the current gap in knowledge, this study aims to investigate the epidemiology of HCV exposure in homeless men in Central Brazil.

## Methods

A cross-sectional study was conducted in homeless men between August and November 2015. The sample consisted of individuals attending four specialized therapeutic communities located in Goiás, Central Brazil, designed with the goal of recovery and reintegration into society for this population. Homeless men were eligible if over 18 years-old. Participants in obvious withdrawal, psychotic break, or psychomotor agitation were considered ineligible.

To calculate the sample size, a statistical power of 80% (β = 20%) with a significance level of 95% (α = 0.05) were considered, and a prevalence of anti-HCV of 8.5% was assumed in the homeless population of São Paulo [11] with design effect correction of 3.0. Therefore, the minimum sample size needed to estimate the prevalence in the study population was 359 participants, which was increased by 10% to correct for loss and refusal, totaling a sample of 395 participants.

Data were collected through interviews using a structured questionnaire containing socio-demographic, behavioral, and clinical risk factors for HCV infection. The questionnaire was based on previously validated studies in populations of homeless and tested in a pilot study [11, 17–22]. Antibodies to HCV (anti-HCV) were detected by using rapid tests on capillary blood collected by finger-stick, as recommended by the World Health Organization and the Ministry of Health of Brazil for vulnerable populations [23, 24]. The test was interpreted ten minutes after the collection and the result was given to the patient during post-test counseling.

The dependent variable of this study was the exposure to HCV. The independent variables included sociodemographic characteristics, factors related to parenteral exposures, and risk behaviors.

### Statistical analysis

Data were analyzed using the statistical program STATA, version 12.0. The *Anderson-Darling* test was used to verify the normality of quantitative variables [25]. Continuous variables were expressed as median and interquartile range (IQR) and categorical variables in absolute and relative frequencies. The prevalence of exposure HCV was estimated with 95% confidence intervals (95.0% CI). Bivariate regression analysis was performed to verify the potential exposure factors associated to HCV. Variables with *p* < 0.10 were included in a Poisson regression model with robust variance and considered statistically significant with values of *p* < 0.05 [26]. Studies indicate that in cross-sectional studies, Poisson models with robust variance are better alternatives than logistic regression. This modeling has been suggested as a good alternative to obtain estimates of adjusted prevalence ratios for potential confounding variables in epidemiological studies. In addition, the use of robust methods for estimating variance in Poisson models corrects the overestimation of variance, and produces adequate confidence intervals, especially when inserted into quantitative variable models [26, 27].

### Ethical aspects

This study was approved by the Ethics Committee of the Clinics Hospital of Federal University of Goiás, under protocol number 1236774. Written informed consent was obtained from all participants. Individuals with positive results were referred to specialized services for further diagnostic confirmation, clinical evaluation, and treatment.

## Results

Overall, 511 individuals were invited to participate in the study; of them, 30 declined, resulting in a response rate of 94.1%, bringing the final study sample to 481. The sociodemographic characteristics of the participants are shown in Table 1. The median age was 36 years (IQR: 36–50), and schooling was eight years (IQR: 6–11). Most of the participants were single (86.3%). The median duration of street experience was 90 days (IQR: 7–1.095).

**Table 1:** Bivariate analysis of potential factors associated with HCV exposure in homeless men in Central Brazil

Variables	Total^a^	HCV		PR^c^ 95.0% CI^d^	*p*
		Pos.^b^	%		
Age (years)	36 (29–45)	45 (36–50)		1.05 (1.00–1.10)	*0.027*
Education (years)	8 (6–11)	9 (6–11)		1.05 (0.89–1.24)	0.528
Time on street (days)^e^	90 (8–1.095)	180 (21–730)		1.02 (0.97–1.07)	0.421
Marital status					
Married	65	2	3.1	1.00	
Others	411	10	2.4	0.79 (0.17–3.60)	0.762
Absence of family life					
No	318	4	1.3	1.00	
Yes	158	8	5.1	4.02 (1.21–13.4)	*0.023*
Prior hospitalization					
No	151	4	2.6	1.00	
Yes	328	8	2.4	0.92 (0.27–3.05)	0.893
Prior blood transfusion^f^					
No	408	8	2.0	1.00	
Yes	72	4	5.6	2.83 (0.85–9.40)	0.089
Tatoo					
No	269	7	2.7	1.00	
Yes	211	5	2.4	0.91 (0.28–2.86)	0.873
Body piercing					
No	359	10	2.8	1.00	
Yes	122	2	1.6	0.58 (0.12–2.68)	0.494
Sharing of personal care items					
No	118	5	4.2	1.00	
Yes	362	7	1.9	0.44 (0.14–1.39)	0.164
Injection drug use					
No	439	4	0.9	1.00	
Yes	42	8	19.0	20.9 (6.29–69.4)	<*0.001*
Alcohol use					
No	120	3	2.5	1.00	
Yes	358	9	2.5	1.00 (0.27–3.71)	0.993
Marijuana use					
No	297	10	3.4	1.00	
Yes	181	2	1.1	0.32 (0.07–1.49)	0.150
Crack use					
No	224	4	1.8	1.00	
Yes	254	8	3.1	1.76 (0.53–5.85)	0.354
Cocaine use					
No	296	7	2.4	1.00	
Yes	182	5	2.7	1.16 (0.36–3.66)	0.789
Condom use					
Always	82	2	2.4	1.00	
Sometimes/never	390	10	2.6	1.05 (0.23–4.79)	0.949
STI history					
No	314	5	1.6	1.00	
Yes	161	7	4.3	2.73 (0.86–8.60)	0.086
Alcohol use before or during sexual intercourse					
Always	211	6	2.8	1.00	
Sometimes/never	269	6	2.2	1.27 (0.41–3.95)	0.674
Illicit drug use before or during sexual intercourse					
Never	137	4	2.9	1.00	
Sometimes/never	341	8	2.3	1.24 (0.37–4.13)	0.721
Sexual intercourse with STI carrier					
No	397	9	2.3	1.00	
Yes	79	3	3.8	1.67 (0.45–6.18)	0.439
Sexual relationship with sex workers					
No	137	1	0.7	1.00	
Yes	343	11	3.2	4.39 (0.56–34.0)	0.156
Sexual relationship with illicit drug users					
No	133	2	1.5	1.00	
Yes	342	10	2.9	1.94 (0.42–8.87)	0.391
Homosexual intercourse					
No	336	9	2.7	1.00	
Yes	144	3	2.1	0.77 (0.21–2.87)	0.706
Number of sexual partners	2 (1–5)	4 (1–10)		1.04 (1.01–1.07)	*0.002*

Note: continuous variables presented as medians and IQR; ^a^Number of valid responses; ^b^Positive; ^c^Prevalence Ratio; ^d^95% confidence interval; ^e^PR estimated for every 180 days of living on the street; ^f^In life

Participants reported risk behaviors for HCV infection, such as alcohol use in the previous 30 days (78.9%), cocaine use (37.1%), crack use (53.1%), and irregular condom use (82.6%). Injecting drug use was reported by 8.7% of the participants.

Anti-HCV markers were detected in 12 of the participants, resulting in a prevalence of 2.5% (95.0% CI: 1.4 to 4.3%). In the bivariate analysis, exposure to HCV was significantly associated with age, education, family life, and injection drug use (*p* < 0.05). These variables, prior blood transfusion (*p* = 0.089), and STI history (*p* = 0.086) were included in the Poisson regression model (Table 1).

In the multiple regression analysis, being without family (adjusted prevalence ratio [aPR]: 4.45; *p* = 0.012), injecting drug use (aPR: 19 2; *p* < 0.001), and STI history (aPR: 3.34; *p* = 0.027) remained as risk factors for HCV infection. HCV prevalence increased by 7% for each year of age (aPR: 1.07; *p* = 0.008) and 7% for each sexual partner in the last year (aPR: 1.07; *p* < 0.001) (Table 2).

**Table 2:** Multiple regression analysis of risk factors associated with HCV exposure in homeless men in Central Brazil

Risk factors	Adjusted^a^ PR^b^ (95.0% CI)^c^	*p*
Age (years)	1.07 (1.01–1.12)	*0.008*
Absence of family life	4.45 (1.39–14.3)	*0.012*
Injection drug use	19.2 (6.01–61.3)	<*0.001*
Blood transfusion history	0.91 (0.19–4.37)	0.913
Number of sexual partners	1.07 (1.04–1.11)	<*0.001*
STI history	3.34 (1.14–9.75)	*0.027*

^a^Adjusted for age, family life, blood transfusion history, injection drug use, number of sexual partners in the last year and STI history; ^b^Prevalence ratio; ^c^95% confidence interval; R^2^: 0.367

## Discussion

This study investigated the HCV prevalence and risk factors among homeless men. To our knowledge, this is the first study conducted in homeless men in Central Brazil. The information in this study provides important data on the extent of HCV in this population, data that can guide actions to prevent and control infection by health services and social assistance to homeless persons. Our findings show a high prevalence of HCV infection in homeless men and an association with age, lack of family life, injection drug use, number of sexual partners, and STI history. In addition, the sample reported high rates of risk behaviors, suggesting that homeless represent a high risk group for pathogens transmitted by the parenteral route and/or sexually.

In this study, the prevalence of anti-HCV antibodies was 2.5% (95.0% CI: 1.4 to 4.3%), higher than that found in the male population of Brazil (1.15%; 95.0% CI: 0.92–1.37%; *χ*2 = 6.823; *p* = 0.009), confirming the vulnerability of homeless men to this infection [28]. However, this rate was lower than the prevalence estimated in homeless men in São Paulo (Southeast Region of Brazil) (9.7%; 95.0% CI: 6.7 to 13.9%) [11]. The difference in prevalence between São Paulo and Goiás can be explained by the different socio-demographic characteristics and risk behaviors of the populations of the two locations. Furthermore, in Brazil, HCV infection has an uneven geographical distribution, with most infected individuals concentrated in the South and Southeast regions, including São Paulo [4].

Considering homeless men in other geographic locations, the prevalence of anti-HCV was lower than that estimated in countries such as Iran (25.1%; 95.0% CI: 21.0–28.5%) [15], England (27.0%; 95.0% CI: 19.0–37%) [9], and USA (31.0%; 95.0% CI: 26.6–35.7%) [5]. Differences in prevalence can be explained, in addition to different endemicity profiles between countries, by variations in the population characteristics and risk behaviors, especially injection drug use. In fact, in this study, the prevalence of injection drug use (8.7%) was low compared to the studies conducted in Iran (27.6%) [15], England (34.0%) [9], and USA (28.2%) [5].

This study found an association between age and HCV exposure, as shown in other studies conducted in vulnerable populations [21, 22, 29–31]. This result may reflect the cumulative risk and multiple parenteral and sexual exposures of these individuals throughout their life [21, 32].

An important determinant of housing on the street includes family problems, drug addiction, and financial difficulties [33], which enhances the social vulnerability of the homeless population. Interestingly, we found an association between the absence of family ties and exposure to HCV. The absence of a family bond can contribute to infections transmitted parenterally and/or sexually, since people without social support and stability may have greater needs as far as individual satisfaction and pursue them through risk behaviors, including injection drug use, unsafe sex, sex work, and multiple sexual partnerships [34–37]. In addition, individuals without social support have greater difficulty accessing health services, which tends to increase the risk of exposure to HCV and other pathogens. Further studies are needed to assess the impact and contribution of social vulnerability in HCV infection in the homeless population.

In both developing and developed countries, most cases of HCV infection occur in people who inject drugs [2]. Worldwide, approximately 60 to 80% of injection drug users are positive for HCV. These individuals are at increased risk for hepatitis C, mainly due to sharing needles and syringes [38]. In this study, injection drug use was strongly linked to HCV exposure, as found in the general population of Brazil [4] and several other studies conducted on homeless people [15, 18, 30, 32, 39]. In addition, among men who reported injection drug use, more than half (57.1%) reported sharing drug paraphernalia (data not shown). These results confirm the efficient transmission of HCV by the parenteral route [29].

Exposure to HCV was associated with STI history and multiple sexual partners. Some studies have shown HCV-RNA detection in semen, vaginal secretions, seminal fluid, saliva, and cervical smear, suggesting the possibility of sexual transmission of the virus, also among homeless men [40, 41]. In addition, factors such as multiple sexual partners, use of illegal drugs before or after sex, inconsistent condom use, and co-infection of some STIs (viral or bacterial) may contribute to sexual transmission of HCV in vulnerable populations [41, 42].

This study has some limitations that should be considered when interpreting the results. The cross-sectional nature does not allow the establishment of cause and effect relationships between HCV exposure and the variables investigated. The data were self-reported, capable of memory and response bias. The study included only men linked to therapeutic communities who consequently do not represent the entire male population living on the streets in Goiás. This study used a positive anti-HCV rapid test as a marker without confirmation of active infection through detection of viral RNA, not differentiating between current or past infection. However, the sensitivity and specificity of the rapid test is high when using blood, serum or plasma (98.4% of sensitivity and 99.7% of specificity), and is a good marker for exposure in vulnerable groups [43].

## Conclusions

In conclusion, this study showed a prevalence of HCV infection in homeless men, higher than the general population of Brazil. Injection drug use was the main risk factor in this population. High rates of risk behaviors, drug use, inconsistent condom use, and multiple sexual partners, were found. The results of this study suggest that this population is at high risk for HCV infection, suggesting the need for effective prevention programs, including health education activities, harm reduction strategies, condom availability, and access to testing and counseling for HCV infection and other parenterally transmitted pathogens. Finally, further studies are needed to verify the actual magnitude of this infection in homeless population in Brazil.

## Abbreviations

aPR: Adjusted prevalence ratio
HCV: Hepatitis C Virus
STI: Sexually transmitted infections
